# Supplementary figures and images for: Age prediction from coronary angiography using a deep neural network: Age as a potential label to extract prognosis-related imaging features
Source: PLoS One. 2022 Oct 27;17(10):e0276928. doi: 10.1371/journal.pone.0276928 (PMC9612526; doi:10.1371/journal.pone.0276928)

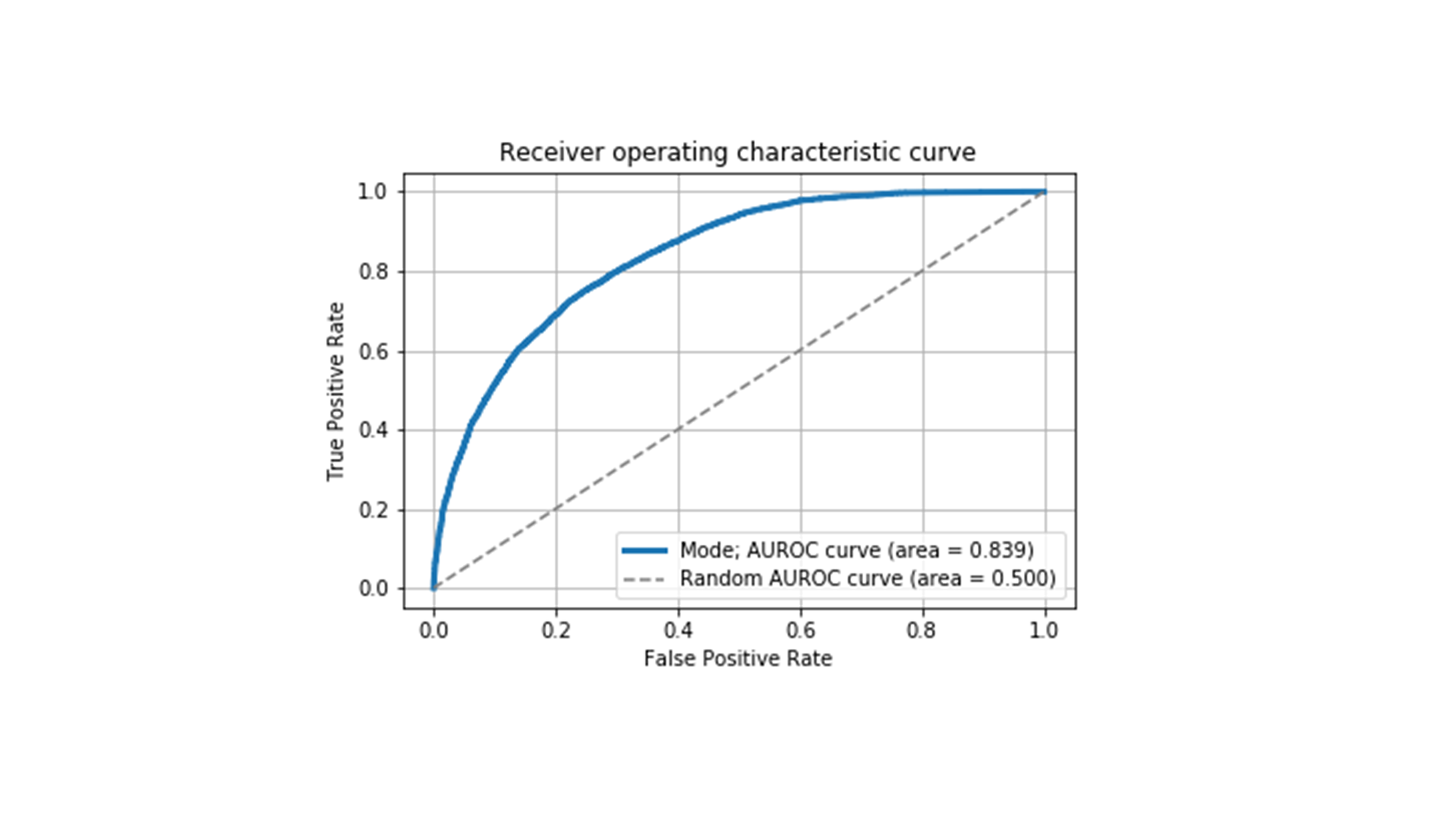

Supplement: S1 Fig — Shown is the ROC curve for age classification (≧ 65 years old) in the test dataset. The overall area under the curve (AUC) was 0.839. (TIF) [file pone.0276928.s001.tif]
